# Mode Effects Between Mobile Web and Telephone Surveys on Patient Experience Scores in South Korea: Secondary Analysis of a Randomized Controlled Trial Under Various Missingness Scenarios

**DOI:** 10.2196/79398

**Published:** 2026-05-14

**Authors:** Young-Geun Choi, Bon Mi Koo, Yeongchae Song, Young Kyung Do

**Affiliations:** 1 Department of Mathematics Education, Sungkyunkwan University, Jongno-gu, Seoul, Republic of Korea; 2 Institute of Pure and Applied Mathematics, Sungkyunkwan University, Jongno-gu, Seoul, Republic of Korea; 3 Institute of Health Policy and Management, Seoul National University Medical Research Center, Jongno-gu, Seoul, Republic of Korea; 4 Department of Health Policy and Management, Seoul National University College of Medicine, 103 Daehak-ro, Jongno-gu, Seoul, 03080, Republic of Korea, 82 2-2072-3124; 5 Review and Assessment Research Department, Health Insurance Review and Assessment Service, Wonju, Gangwon, Republic of Korea

**Keywords:** patient experience survey, telephone survey, mobile web survey, mode effect, missing data, sensitivity analysis

## Abstract

**Background:**

Patient experience surveys are essential tools for assessing health care quality, yet the potential influence of survey mode on patient experience scores remains understudied. This study investigates the mode effects between mobile web and telephone surveys within South Korea’s Patient Experience Assessment.

**Objective:**

This study aimed to examine the presence and extent of the mode effects of mobile web versus telephone surveys on patient experience scores. The primary outcome was defined as the total score across all 21 survey items, rescaled to 0‐100.

**Methods:**

This is a secondary analysis using experimental data from a parallel-group randomized controlled trial involving 3200 patients (adults aged ≥19 years, hospitalized >1 day, discharged 2‐56 days before the survey) from 4 general hospitals between October and November 2022, equally allocated to telephone and mobile web survey modes. An independent survey company generated the random allocation sequence using computer-generated random numbers and assigned participants to the survey modes. Due to the nature of the intervention, blinding of participants, interviewers, and outcome assessors was not feasible after assignment. We calculated unadjusted score differences among respondents and estimated adjusted differences accounting for nonresponse using inverse probability weighting (IPW) and multiple imputation (MI) under the missing-at-random assumption. Sensitivity analyses, using the delta-adjustment method based on the missing-not-at-random assumption, assessed robustness to departures from the missing-at-random assumption. Subgroup analyses by sex, age group, and field of care were also conducted.

**Results:**

Of 3200 patients randomized (1600 per mode), 878 completed the survey (520 mobile web and 358 telephone). Analyses included all randomized participants (n=3200), with nonresponse addressed through IPW and MI. No adverse events were reported in this survey-based study. The total patient experience score was significantly lower in the mobile web group (mean 81.5, SD 16.4) than in the telephone group (mean 84.9, SD 14.3; unadjusted difference –3.41 points, 95% CI –5.51 to –1.31; IPW-adjusted –4.11, 95% CI –6.17 to –2.04; MI-adjusted –4.59, 95% CI –7.45 to –1.73). Similar patterns were observed across most subdomains. Subgroup analyses revealed consistent mode effects across different demographic categories. Sensitivity analyses using the delta-adjustment method confirmed the robustness of these findings under various missing data scenarios.

**Conclusions:**

Mobile surveys may yield substantially lower patient experience scores than telephone surveys. Unlike previous studies, our study analyzes randomized experimental data under various missingness scenarios and provides evidence that this mode effect is unlikely to be attributable to analytical methods or heterogeneity in respondent characteristics between the 2 survey administration modes. Accordingly, caution is warranted when comparing patient experience scores obtained from traditional telephone surveys with those from mobile surveys. Methodologically, our sensitivity analysis approach provides a robust framework for assessing and addressing potential nonresponse bias in patient experience assessments.

## Introduction

### Background and Rationale

Incorporating the patient’s perspective in assessing health care quality is gaining prominence globally, with various countries and international organizations highlighting its significance [1-4]. Consequently, there is a growing effort to systematically and consistently collect data, focusing not only on patient-centered health outcomes but also on patient experience [5,6]. Patient experience concerns the nontechnical aspect of health care quality, as illustrated by the communication between inpatients and their health care providers throughout their hospital stay [7]. While measuring and publicly reporting patient experience ultimately aims to enhance patient experience across the health system, individual hospitals are also key actors in this process [8]. These hospitals are keenly interested in the publicly reported results of patient experience surveys, as these results may significantly affect their reputation and competitiveness in the health care market. A positive correlation has been noted between patient experience and hospital profitability [9]. The stakes are heightened when the results of patient experience surveys are used for third-party payers to adjust hospital reimbursement, aligning with the notion of value-based care [10,11]. For these reasons, the imperative of ensuring a fair comparison becomes a critical methodological consideration in generating hospital-level patient experience results. Nevertheless, hospitals vary in many ways, most notably in terms of different patient characteristics, which can affect hospitals’ patient experience scores [12]. This challenge is further compounded by the effects of survey administration mode on who chooses to participate and who does not, as well as on how respondents answer, thereby introducing the central issue of missing data due to nonresponse [12]. As web-based survey administration gains popularity alongside traditional modes such as mail and telephone surveys in patient experience initiatives, a growing challenge is effectively accounting for the influence of these diverse survey modes on differential missingness and the resulting bias in estimating causal mode effects.

The term “mode effect” refers to the difference between the measured score obtained through one survey mode and the counterfactual score that would have been measured if the survey had been conducted by another mode [13,14]. Estimating mode effects, therefore, necessitates an experimental study and careful statistical adjustments based on appropriate assumptions [15]. In the context of patient experience surveys, several countries have conducted rigorous experiments to understand these effects. In the United States, the Hospital Consumer Assessment of Healthcare Providers and Systems (HCAHPS) survey uses multiple modes: mail, telephone, mail with telephone follow-up, and interactive voice response (IVR). An experimental study in 2006 reported that the telephone and IVR modes generally yielded more positive evaluations compared to mail surveys. In England, the National Health Service Adult Inpatient Survey has been exploring the transition to a mixed-mode methodology. A pilot study conducted in 2019 tested two experimental push-to-web approaches against the traditional paper-based method [16]. The study found that online responses tended to be more negative than paper responses.

Statistically, a critical step in estimating mode effects is adjusting for selective nonresponse. The missing-at-random (MAR) assumption is commonly used, positing that score distributions for respondents and nonrespondents remain consistent after accounting for survey mode and other variables influencing both mode selection and scores. If valid, this assumption allows for unbiased estimation of true mode effects by adjusting for respondent composition. Elliott et al [12] reported adjusted mode effects under MAR, while Ipsos MORI (2020) [16] presented unadjusted estimates. However, growing evidence suggests limitations of the MAR assumption in estimating survey mode effects. Multiple studies [17-23] indicate that late responders and nonresponders often report less favorable health care experiences than early responders. Consequently, assessing the robustness of MAR-based results is crucial. Experts recommend sensitivity analyses by re-estimating mode effects under missing-not-at-random (MNAR) scenarios, where distributions of observed and unobserved outcome scores may differ even after adjusting for response probability factors [24-26]. MNAR analyses, while free from the MAR assumption, require additional assumptions about nonrespondents’ score distributions. Two common MNAR strategies are the pattern mixture model [27] and the sample selection model [28]. The “delta-adjustment” approach based on the pattern mixture model is often preferred for its fewer restrictive assumptions and easily interpretable results [26,29,30]. Combining MAR-based primary analysis with MNAR-based sensitivity analysis can yield more robust mode effect estimates by examining their stability across different missing data assumptions.

### Objectives

This study examines mode effects between telephone and mobile web surveys in South Korea’s Patient Experience Assessment (PXA). Our previous study [31] focused on the influence of survey mode on response rates in the PXA. This study, a companion to our previous work, examines the impact of survey mode on patient experience scores, a distinct research question using different outcome measures and analytical approaches. Specifically, we aim to: (1) quantify differences in patient experience scores between telephone and mobile web surveys, (2) examine adjusted differences accounting for potential nonresponse bias under the MAR assumption, and (3) conduct sensitivity analyses using the delta adjustment to assess the robustness of MAR-based findings under the MNAR assumption. By addressing these objectives, we aim to provide valuable insights into the policy considerations for the PXA in South Korea and to contribute to the broader international discourse on survey methodology in patient experience surveys. As this study involves survey administration without clinical interventions, no harm to participants was anticipated.

## Methods

### Trial Design, Trial Setting, and Eligibility Criteria

This study is a secondary analysis of a randomized controlled trial conducted by our research team at 4 general hospitals in South Korea (2 in Seoul, 1 in Ulsan, and 1 in Gyeongju). Eligible patient lists were obtained from participating hospitals from early October 2022, and the survey was conducted from October 24 (first participant enrolled) to November 18, 2022. The trial was registered retrospectively (December 23, 2025). This study adheres to the CONSORT 2025 (Consolidated Standards of Reporting Trials) guidelines for reporting randomized trials (Checklist 1) [32]. The sample selection in the experiment followed the current PXA framework in South Korea, with minor modifications. Specifically, inclusion criteria were: (1) adults aged 19 years and older, (2) inpatients for >1 day, and (3) discharged 2‐56 days before survey participation. Exclusion criteria included patients from day clinics, palliative care, pediatrics, neuropsychiatry, and those without completed personal information consent forms. Hospital staff identified eligible patients. The study recruited a total of 4800 patients from 4 participating general hospitals. To ensure representativeness of the subpopulations, stratified sampling was performed based on sex (male and female), age group (19‐39, 40‐59, 60‐69, and 70 years and older), field of care (medical and surgical and others), and hospital of origin (A, B, C, and D). The sampled patients were randomly assigned to 1 of 3 survey modes (telephone, mobile web, and mixed) in a 3-arm parallel-group exploratory design with a 1:1:1 allocation ratio, with individual patients as the unit of randomization. As the goal of our study is to accurately estimate the mode effect between telephone and mobile web surveys, we used the data from 3200 subjects from the telephone and mobile web survey modes (1600 for each). A total of 878 participants (358 for the telephone mode and 520 for the mobile web mode) provided responses to all questions.

### Randomization and Blinding

Simple randomization was used, with no blocking or stratification applied. An independent survey company generated the random allocation sequence using computer-generated random numbers (via the NEWID() function in Microsoft SQL Server) and assigned participants to the survey modes. No other parties, including the research team, hospital staff who enrolled participants, interviewers, and participants themselves, had access to the allocation sequence prior to assignment. Due to the nature of survey mode interventions, blinding was not feasible after assignment.

### Intervention and Comparator

Survey procedures varied by mode. In the telephone survey, trained interviewers, who received standardized training on the PXA survey protocol prior to the study, made up to 5 daily call attempts, following a standardized protocol. In the mobile web survey, participants received up to 5 survey link messages via an official messenger app. The survey process involved clicking through domain-specific screens and submitting responses. The mobile survey interface was optimized based on usability testing. All participants were informed about the survey beforehand. Each mode followed a similar sequence of identity verification, information provision, questionnaire administration, and conclusion.

### Patient and Public Involvement

There was no patient or public involvement in the design, conduct, or reporting of this trial.

### Changes to Trial Protocol

No changes were made to the trial protocol after commencement. Subgroup analyses and sensitivity analyses under MNAR scenarios were not prespecified in the original protocol but were conducted post hoc (see Statistical methods).

### Outcomes

The prespecified primary outcome was the total patient experience score, calculated as the sum of responses across all 21 questions of the PXA questionnaire. The questionnaire comprises six domains: nurse (Q1–Q4, 4 items), doctor (Q5–Q8, 4 items), medication and treatment (Q9–Q13, 5 items), hospital environment (Q14–Q15, 2 items), patient’s rights (Q16–Q19, 4 items), and overall ratings (Q20–Q21, 2 items). Most items use a 4-point Likert scale (1=never or not at all to 4=always or very much), while Q13 uses a binary (yes or no) format, and the 2 overall rating items (Q20–Q21) use a 0‐10 scale. All item scores were rescaled to a maximum of 100 points per item before summation, adhering to the current policy convention in the Korean PXA. The prespecified secondary outcomes comprised the sum scores for each of the 6 domains, also rescaled to 100 points. The timepoint of outcome assessment was 2 to 56 days after hospital discharge, corresponding to the survey administration period. Outcomes were self-reported by participants in the mobile web group and collected by the trained interviewers in the telephone group.

### Harms

As this study involved only survey administration (telephone calls or mobile web survey links) without any clinical interventions, no physical or clinical harm was anticipated. Therefore, systematic harm monitoring was not conducted.

### Sample Size

Sample size calculation was based on detecting a 1-point difference in patient experience scores (SD=2, 80% power, α=.05) using G*Power (version 3.1; HHU), requiring 64 respondents per group. Assuming a 10% response rate, this necessitated recruiting 640 participants per mode. To ensure sufficient power for planned subgroup analyses, the study recruited 1600 per mode. No interim analyses were planned or conducted for this study.

### Statistical Methods

As a basic characteristic, response rates by survey mode and study participants’ characteristics (sex, age group, field of care, and hospital of origin) were reported as counts and percentages. The chi-square test was used to identify statistically significant differences in response rates between modes.

For the analysis of the overall score and six subscores, an unadjusted average score by mode was calculated from respondents who completed the survey. The unadjusted score difference between the two modes was also reported. As the main analysis, an adjusted difference was estimated under the MAR assumption, ie, the outcome distribution of the nonrespondent group was assumed to be the same as that of the respondent group for each stratum of mode, sex, age group, field of care, and hospital of origin. Both inverse probability weighting (IPW) and multiple imputation (MI) methods were used for the adjustment. The IPW-adjusted difference calculates the weighted mean difference between the respondents in the two modes, where the weights adjust for nonresponse so that the respondent sample better represents the full randomized population. The propensity score model for the weights used logistic regression to predict response probability from the aforementioned stratification variables. Variance for IPW estimates was calculated using robust sandwich estimators to account for weight estimation uncertainty. The MI-adjusted difference calculates the unweighted difference between the two modes from the full dataset where the missing scores of the nonrespondents are imputed by regression. Specifically, we used Bayesian linear regression with the stratification variables as predictors, generating 50 imputed datasets. Variance was obtained by applying Rubin’s rules, which properly combine within- and between-imputation variability. It is worth noting that both methods are complementary, as they rely on different statistical assumptions; IPW relies on the assumption that the probability of response for each stratum was accurately estimated, while MI assumes that the mean outcome (score) for each stratum was accurately estimated. For clarity, the target estimand is formally defined in Supplementary Material A.1 (Multimedia Appendix 1), representing the average causal effect of survey mode on patient experience scores among our eligible patient population. It is known that both the IPW and MI-adjusted difference unbiasedly estimate the target estimand under the MAR assumption.

For subgroup analyses, the same outcome analyses were reconducted across subpopulations categorized by sex, age group, and field of care, as these are known to be associated with both survey response patterns and patient experience scores [12,31].

To check the robustness of the results against the MAR assumption, we conducted sensitivity analyses by the delta-adjustment method under the pattern mixture model that assumes the MNAR scenario. The delta-adjustment method is popular for the MAR-sensitivity analyses due to its simplicity for interpretation and implementation [26,29,30]. Specifically, the adjusted score difference between the 2 mode groups was recalculated, assuming that the mean outcomes of the nonrespondent telephone survey group and the nonrespondent mobile web survey group are shifted by δ_1_ and δ_2_, respectively, compared to their respondent counterparts. The values of δ_1_ and δ_2_ must be specified by the analyst, and vary from −5 to 5 points in our study. A detailed description and the model formula are deferred to Supplement Material A (Multimedia Appendix 1). For example, if δ_1_ is −3, the nonrespondent telephone survey group is assumed to have scores that are 3 points lower than the respondent telephone survey group. The case where δ_1_=δ_2_=0 coincides with the MAR assumption. The delta-adjustment method is available via the MI method, which naturally incorporates the shift in mean outcomes.

The primary analysis examining mode effects on patient experience scores was prespecified in the original study protocol. Subgroup analyses and sensitivity analyses using the delta-adjustment method were conducted post hoc to further assess the robustness and generalizability of the primary findings.

All statistical analyses were performed using R (version 4.4.1; R Foundation for Statistical Computing). A *P* value of <.05 was considered statistically significant.

### Ethical Considerations

An application was submitted to the Seoul National University Hospital’s Institutional Review Board (IRB No. H-2207-134-1342), as well as the IRBs of 3 other general hospitals (IRB Nos. 2022‐1284, Asan Medical Center; UUH2022-07-043, Ulsan University Hospital; and 110757‐202207-HR-03-04, Dongguk University Gyeongju Hospital). All these boards approved the study, which involved patients discharged from these hospitals. It is important to note that the study only included patients who, at the time of admission, had agreed to the use of their personal information by signing the respective hospital’s consent form. This was a part of the exclusion criteria to guarantee voluntary participation. All participant data were deidentified before analysis, with only study identification numbers used to link survey responses. No compensation was provided to participants for survey completion. This manuscript does not include any images or information that could identify individual participants.

Clinical Research Information Service KCT0011374 was registered retrospectively on December 23, 2025 (first participant enrolled: October 24, 2022). The trial protocol, the full statistical analysis plan, and preliminary results are accessible through this registry.

## Results

### Participant Flow, Recruitment, and Baseline Data

**Figure 1.**
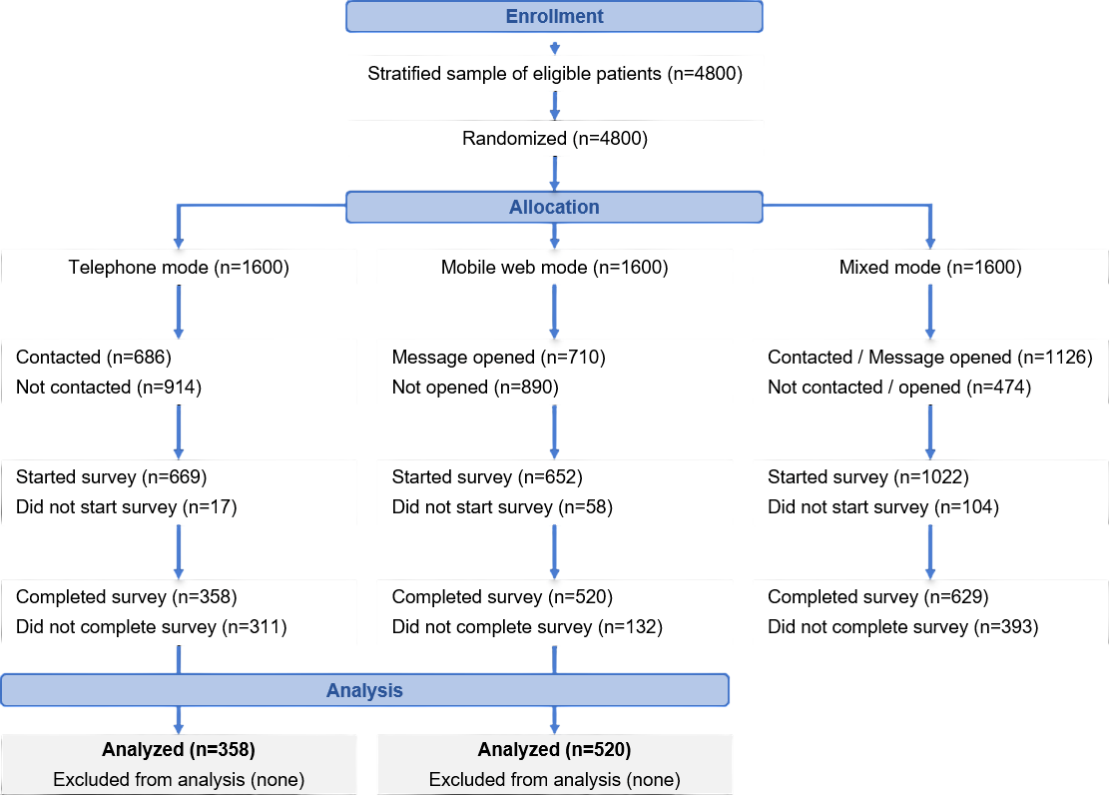
CONSORT (Consolidated Standards of Reporting Trials) flow diagram of the study participants. All 4800 participants were randomly allocated to one of three survey modes (n=1600 each). To estimate the mode effects accurately, the current analysis included only the telephone (n=358) and mobile web (n=520) groups (total n=878). The mixed-mode group was excluded in this comparative analysis.

Of the 3200 participants randomized to telephone and mobile web modes, 878 completed the survey (Figure 1 for detailed participant flow). Both interventions were delivered as planned, with no deviations from the protocol. No concomitant interventions that could have affected the outcomes were identified. Table 1 presents response rates by survey mode and study participants’ characteristics. The overall response rate was 27.4% (878/3200), with a significantly higher rate in the mobile web group (520/1600, 32.5%) compared to the telephone group (22.4%, 358/1600; *P*<.001). Notably, response rates varied across sex, age group, field of care, and hospital of origin, with significant differences between the 2 modes in most categories. This heterogeneity in respondent composition reaffirms the need for appropriate statistical adjustment methods under the MAR or MNAR scenarios to obtain unbiased estimates of mode effects on survey outcomes.

**Table 1.** Response rates of the study sample by survey mode and participant characteristics^a^.

Characteristics	All (n=3200)	Telephone (n=1600)	Mobile web (n=1600)	*P* value
	Respondents/randomized (%)	Respondents/randomized (%)	Respondents/randomized (%)	
Total	878/3200 (27.4)	358/1600 (22.4)	520/1600 (32.5)	<.001
Sex				
Male	445/1583 (28.1)	195/799 (24.4)	250/784 (31.9)	<.001
Female	433/1617 (26.8)	163/801 (20.3)	270/816 (33.1)	
Age group (years)				
19‐39	115/414 (27.8)	31/189 (16.4)	84/225 (37.3)	<.001
40‐59	359/1030 (34.9)	128/525 (24.4)	231/505 (45.7)	
60‐69	253/878 (28.8)	116/439 (26.4)	137/439 (31.2)	
70+	151/878 (17.2)	83/447 (18.6)	68/431 (15.8)	
Field of care				
Medical	310/1268 (24.4)	123/612 (20.1)	187/656 (28.5)	<.001
Surgical and other	568/1932 (29.4)	235/988 (23.8)	333/944 (35.3)	
Hospital of origin				
A	314/1000 (31.4)	136/500 (27.2)	178/500 (35.6)	<.001
B	288/1002 (28.7)	127/502 (25.3)	161/500 (32.2)	
C	158/666 (23.7)	50/334 (15)	108/332 (32.5)	
D	118/532 (22.2)	45/264 (17)	73/268 (27.2)	

a Data from a randomized controlled trial conducted in October–November 2022 at four general hospitals in South Korea. A total of 3200 adult inpatients (discharged 2–56 d prior) were randomly allocated to telephone (n=1600) or mobile web (n=1600) survey modes. The chi-square test was used to compare response rates between modes. Response rate (%) is defined as the number of complete responses divided by the number of patients randomized to each mode.

### Numbers Analyzed, Outcomes, and Estimation

Table 2 presents the unadjusted and adjusted estimates for mode effects on the total score and domain scores. The unadjusted estimates were based on the 878 respondents (358 telephone and 520 mobile web), while adjusted estimates using IPW and MI incorporated all 3200 full randomized participants to account for nonresponse. The primary outcome, the total score, was significantly lower in the mobile web group compared to the telephone group, with an unadjusted difference of −3.41 points (95% CI −5.51 to −1.31; *P*=.001). This difference remained significant after adjusting for missing data using the IPW method (−4.11 points; 95% CI −6.17 to −2.04; *P*<.001) and the MI method (−4.59 points; 95% CI −7.45 to −1.73; *P*=.007). Among the secondary outcomes, the hospital environment domain score showed the largest difference between the 2 modes, with an unadjusted difference of −6.63 points (95% CI −9.30 to −3.96; *P*<.001) and adjusted differences of –8.36 points (IPW; 95% CI –10.95 to −5.77; *P*<.001) and −7.81 points (MI; 95% CI −11.42 to −4.20; *P*=.001). The scores in the nurse, doctor, and medication and treatment domains also showed significantly lower scores in the mobile web group compared to the telephone group, both in the unadjusted and adjusted analyses. The patient’s rights domain score showed a marginally significant difference in the IPW-adjusted analysis (−2.60 points; 95% CI −5.16 to −0.04; *P=*.05) but not in the unadjusted (−2.05 points; 95% CI −4.61 to 0.52; *P*=.12) or MI-adjusted analyses (−2.92 points; 95% CI −6.93 to 1.09; *P*=.15). This inconsistency between IPW and MI adjustment may be due to the difference in underlying assumptions of the adjustment methods: IPW assumes correct specification of the response propensity model, while MI relies on appropriate modeling of the outcome distribution. Finally, the overall rating score did not show a statistically significant difference between the 2 modes in either the unadjusted or adjusted analyses.

We then assessed whether the mode effect on the total score differed across levels of sex, age group, and field of care (Tables S1–S7 in Multimedia Appendix 1). For the total score and the 6 domain scores, the *P* values for interaction were generally greater than .05 across the subgroups, indicating that the mode effect was consistent within each subgroup and did not significantly differ between subgroups.

**Table 2.** Unadjusted and adjusted estimates for mode effects on the total score and domain scores.^a^

		Telephone mean (SD)	Mobile mean (SD)	Unadjusted^b^		IPW–Adjusted^b^		MI–Adjusted^b^	
Outcomes				Diff.^c^ (95% CI)	*P* value	Diff.^c^ (95% CI)	*P* value	Diff.^c^ (95% CI)	*P* value
Primary outcome									
Total		84.92 (14.34)	81.51 (16.35)	−3.41 (−5.51 to −1.31)	.001	−4.11 (−6.17 to −2.04)	<.001	−4.59 (−7.45 to −1.73)	.007
Secondary outcomes									
Nurse		89.40 (15.20)	86.02 (17.06)	−3.38 (−5.58 to −1.18)	.003	−4.44 (−6.60 to −2.29)	<.001	−4.79 (−7.80 to −1.79)	.005
Doctor		82.51 (18.79)	78.43 (19.93)	−4.08 (−6.71 to −1.46)	.002	−4.97 (−7.56 to −2.39)	<.001	−5.21 (−8.77 to −1.65)	.01
Medication and treatment		85.08 (16.69)	81.98 (18.78)	−3.10 (−5.52 to −0.68)	.01	−3.93 (−6.33 to −1.52)	.001	−4.48 (−7.77 to −1.19)	.01
Hospital environment		86.13 (18.28)	79.50 (20.85)	−6.63 (−9.30 to −3.96)	<.001	−8.36 (−10.95 to −5.77)	<.001	−7.81 (−11.42 to −4.20)	.001
Patient’s rights		80.81 (19.83)	78.76 (18.47)	−2.05 (−4.61 to 0.52)	.12	−2.60 (−5.16 to −0.04)	.05	−2.92 (−6.93 to 1.09)	.15
Overall rating		85.22 (16.94)	84.12 (19.10)	–1.10 (−3.56 to 1.36)	.38	0.45 (–1.99 to 2.89)	.72	–0.77 (–3.92 to 2.37)	.63

a Results from a randomized controlled trial of 3200 adult inpatients from four general hospitals in South Korea (October-November 2022), with 878 complete responses (358 telephone and 520 mobile web).

b Unadjusted estimates are based on complete responses. IPW (inverse probability weighting) and MI (multiple imputation) adjusted estimates account for potential nonresponse bias under the missing-at-random (MAR) assumption.

c “Diff.” means difference in mean scores (mobile web – telephone). Negative differences indicate lower scores in the mobile web survey compared to the telephone survey. All scores are scaled to a maximum of 100 points.

### Harms

No adverse events or unintended effects were identified in either the telephone or mobile web survey group during the study period. This was anticipated, as the intervention involved only survey administration without any clinical procedures. No complaints or adverse reactions were reported during the survey process.

### Ancillary Analyses

**Figure 2.**
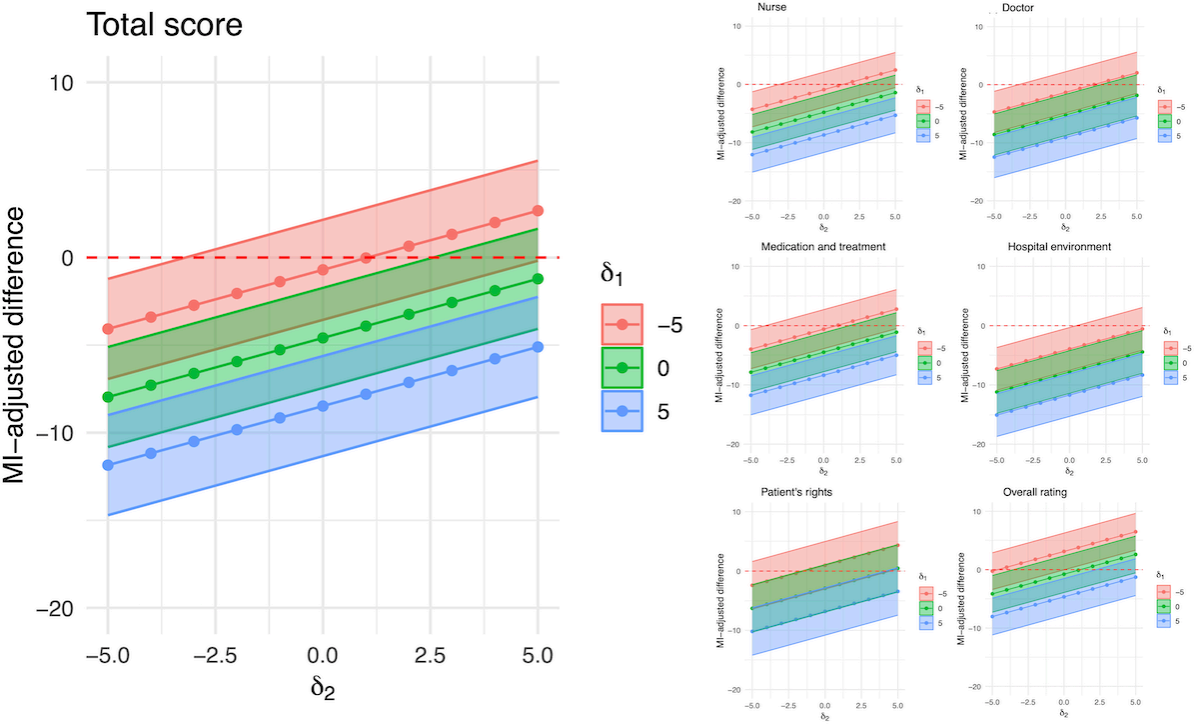
Estimated mode effects by the delta-adjustment method under a missing-not-at-random (MNAR) scenario, varying values of δ_1_ and δ_2_. MI: multiple imputation.

A sensitivity analysis under MNAR scenarios was conducted to assess the robustness of the MAR assumption by varying the values of δ_1_ and δ_2_, which represent the assumed differences in mean scores between nonrespondents and respondents for the telephone and mobile web survey groups, respectively. Figure 2 depicts the estimated difference of scores (=mobile web – telephone) for given values of δ_1_ and δ_2_. For instance, δ_1_=0 and δ_2_=−2.5 represents a scenario where telephone nonrespondents would have had similar patient experience scores to telephone respondents, while mobile web nonrespondents would have had scores 2.5 points lower than mobile web respondents; under this scenario, the mode effect remains substantial at −6.84 points (95% CI −9.13 to −4.55) for the total score. The figure reveals that as δ_1_ increases and δ_2_ decreases, the mode effect between telephone and mobile web surveys becomes more pronounced in the negative direction. In other words, if nonrespondents in the telephone survey are assumed to give higher scores than respondents, while nonrespondents in the mobile web survey are assumed to give lower scores than respondents, the negative mode effect is amplified. Conversely, when δ_1_ decreases and/or δ_2_ increases, the mode effect is attenuated, suggesting that if nonrespondents in the telephone survey are assumed to give lower scores than respondents, while nonrespondents in the mobile web survey are assumed to give higher scores than respondents, the difference between the 2 modes is reduced. Overall, most of the plots for the total score and domain scores show a negative mode effect, consistent with the IPW and MI results presented in Table 2. The telephone survey would only yield better scores than the mobile web survey under the artificial assumption that δ_1_ is −5 and δ_2_ is positive. Even if δ_1_ is set to 0, δ_2_ would have to be less than 2.5 for the telephone survey to show higher scores than the mobile web survey across most domains. These findings suggest that, even under the MNAR scenarios, the possibility of the mobile web survey yielding higher scores than the telephone survey is highly unlikely, except under some unusual combinations of δ_1_ and δ_2_ values.

## Discussion

### Interpretation

In accordance with our study objectives, we examined mode effects between mobile web and telephone surveys on patient experience scores under various missing data assumptions. The main analyses consistently showed that, under the widely accepted MAR assumption, the mobile web survey yielded significantly lower scores compared to the telephone survey for both the total score and almost all domain scores. The sensitivity analysis under the MNAR assumption, our third objective, further supported this finding, revealing that in most plausible scenarios, the mobile web survey scores remained lower than the telephone survey scores, even when considering potential departures from the MAR assumption. Only under extreme and unlikely scenarios where the nonrespondent telephone survey group was assumed to have substantially lower scores than the respondent telephone survey group (eg, δ_1_=−5) while simultaneously assuming the nonrespondent mobile web survey group had higher scores than the respondent mobile web survey group (eg, δ_2_>0), did the pattern reverse. These findings highlight the robustness of the observed mode effect, suggesting that the lower scores in the mobile web survey are not merely an artifact of the MAR assumption. Moreover, the randomized controlled design ensures that these mode effects are not attributable to confounding by respondent characteristics between the 2 survey modes.

### Findings in Relation to Other Evidence

Our findings are consistent with the existing literature indicating that interviewer-led surveys, whether conducted face-to-face or via telephone, often yield more positive responses to scale questions compared to self-administered surveys, such as those completed by mail or online [12,33-38]. This pattern extends well beyond health care surveys. Leeuw [37] meta-analysis established that interviewer-led modes consistently elicit more socially desirable responses than self-administered modes. Dillman et al [34] experimentally demonstrated that aural modes (telephone and IVR) produced significantly more extreme positive responses to satisfaction questions than visual modes (mail and web). A large-scale experiment by the Pew Research Center in 2015 [38], randomizing respondents across 60 questions, reported an average telephone–web difference of approximately 5 percentage points, with the largest gaps on topics susceptible to social desirability. Together, these findings indicate that the tendency for interviewer-led surveys to yield more positive responses is robust across diverse topics, populations, and cultural contexts.

In the context of patient experience surveys, our findings align with Elliott et al HCAHPS study [12], showing consistent mode effects across different cultural contexts and health care systems. Both studies found that telephone surveys yielded more positive evaluations than self-administered modes (mail or mixed in HCAHPS, mobile web in ours). Interestingly, both studies found a significant mode effect for the Nurse domain and a nonsignificant effect for the Overall ratings domain. These similarities across different health care systems, languages, and survey designs further support the robustness of mode effects and highlight the importance of considering these effects in patient experience assessments, regardless of the specific context.

The observed mode effect can be attributed to various factors [33-35]. First, the social setting of the survey itself can influence responses: interview modes involve a social interaction between respondent and interviewer, which is absent in self-administered modes, potentially affecting response patterns [33]. Second, social desirability bias may lead respondents to provide more socially acceptable answers in the presence of an interviewer, resulting in an overreporting of desirable behaviors and higher scores in telephone surveys [33,34]. Third, acquiescence bias, or the tendency to agree with statements or questions when in doubt, may be more prominent in interview situations [33,34]. Fourth, the recency effect—where respondents tend to select the last option presented in an auditory mode—can lead to higher selection of positive top-box categories in telephone surveys, as these are typically read last [34,35]. These factors may collectively contribute to the higher scores observed in the telephone survey mode compared to the self-administered mobile web mode across most domains.

### Research Implications

The sensitivity analysis in this study helps to assess the robustness of results against potential deviations from the MAR assumption. Multiple imputation with delta adjustment offers a method for imputing missing data under various MNAR scenarios [26,29,30]. By systematically varying the delta parameter, researchers can explore how their conclusions might change under different missing data mechanisms. This approach is particularly valuable when nonresponse is prevalent [39-41], as in the context of patient experience surveys, where mode-specific nonresponse patterns may introduce bias that standard MAR-based methods cannot detect.

While our analysis assumed a constant shift parameter (ie, a fixed degree of departure from MAR) for all individuals within each survey mode group, one could explore the possibility of varying delta values across different individuals in the same group. For instance, in an HIV longitudinal study [40], for participants without actual outcomes (serological HIV test results), the researchers assumed different delta values based on the participants’ self-reported HIV status. This approach allowed for a more nuanced sensitivity analysis, accounting for potential differences in the missing data mechanism among subgroups within the same study group.

### Policy Implications

Our results have 2 important policy implications for South Korea’s PXA. First, by demonstrating that survey modes have substantial effects on patient experience scores, our findings imply that mixing different modes in South Korea’s PXA would introduce additional methodological challenges for making fair comparisons across hospitals. Efforts to improve response rates in the PXA have included introducing a mobile web survey either in place of or alongside the existing telephone survey [31]. However, mixing 2 different modes would have required adjustments for mode effects. In 2023, the PXA survey switched to a single mode of mobile web, thereby eliminating the need to account for mode effects between survey methods [42]. Second, caution is still needed when comparing PXA scores between 2023 and previous years. Based on our findings, PXA scores in 2023 are expected to show a decline, all else being equal, due to the mode effect of the mobile web survey compared to the telephone survey used in prior years. Policymakers, the general public, and the media should anticipate lower scores in 2023 due to this mode effect and should not interpret this potential decline as an indication of worsening patient experience over time.

### Limitations

This study has several limitations. First, using data from patients at 4 specific hospitals in South Korea may limit the generalizability of the findings to other health care and cultural settings. Second, because the study compares mobile web with telephone survey modes, the results are less generalizable to populations with relatively low mobile device usage. Third, the use of sum scores for analysis, while consistent with current policy practices, may obscure nuances in individual item responses that could be affected differently by survey mode. Fourth, our study cannot identify or disentangle the specific mechanisms underlying the observed mode effects, including social interaction effects, social desirability bias, acquiescence bias, and the recency effect.

### Conclusions

Mobile surveys may yield substantially lower patient experience scores than telephone surveys. Unlike previous studies, our study analyzes randomized experimental data under various missingness scenarios and provides evidence that this mode effect is unlikely to be attributable to analytical methods or heterogeneity in respondent characteristics between the 2 survey administration modes. Accordingly, caution is warranted when comparing patient experience scores obtained from traditional telephone surveys with those from mobile surveys. Methodologically, our sensitivity analysis approach provides a robust framework for assessing and addressing potential nonresponse bias in patient experience assessments.

## Supplementary material

10.2196/79398 Multimedia Appendix 1 Statistical analysis details and additional results.

10.2196/79398 Checklist 1 CONSORT 2025 checklist.

10.2196/79398 Checklist 2 CONSORT e-HEALTH (V1.6.1) checklist.

10.2196/79398 Checklist 3 Items to include when reporting a randomized trial in a journal or conference abstract.

## Abbreviations

CONSORT: Consolidated Standards of Reporting Trials
HCAHPS: Hospital Consumer Assessment of Healthcare Providers and Systems
IPW: inverse probability weighting
IVR: interactive voice response
MAR: missing-at-random
MI: multiple imputation
MNAR: missing-not-at-random
PXA: Patient Experience Assessment
