# Supplementary material for: Mode Effects Between Mobile Web and Telephone Surveys on Patient Experience Scores in South Korea: Secondary Analysis of a Randomized Controlled Trial Under Various Missingness Scenarios
Source: J Med Internet Res. 2026 May 14;28:e79398. doi: 10.2196/79398 (PMC13175444; doi:10.2196/79398)
Supplement: Multimedia Appendix 1 [file jmir-v28-e79398-s001.docx]

Supplementary Materials

Supplementary Material A. Statistical analysis details

Supplementary Material B. Additional results

Supplementary Material A. Statistical analysis details

A.1. Target estimand

Our target estimand is the average causal effect of survey mode on patient experience scores among the eligible patient population. Specifically,

Target population: Adults aged 19 or older who were discharged from general hospitals in South Korea after at least one overnight stay, excluding patients from day clinics, palliative care, pediatrics, neuropsychiatry, and those without completed personal information consent forms.

Estimand: $E[Y^{\left( mobile \right)} - Y^{(telephone)}]$, where $Y^{\left( mode \right)}$ represents the potential outcome (patient experience score on a 0–100 scale) that would be observed if all eligible patients were surveyed using that mode. This estimand represents the average treatment effect (ATE) of mobile web survey versus telephone survey on patient experience scores. Due to randomization, the difference in observed means between modes provides an unbiased estimate of this causal effect among respondents, provided that either all patients respond or the non-response pattern is independent of the survey modes. However, if there is differential non-response between modes, this necessitates adjustment to recover the ATE for the full eligible population, which is the focus of our IPW and MI analyses under the MAR assumption and our sensitivity analyses under MNAR scenarios.

A.2. Detailed description of the delta-adjustment method

The delta adjustment method assesses the sensitivity of our results to potential departures from the Missing-at-Random (MAR) assumption.

Recall that under MAR, the basis for our initial multiple imputation (MI), we assumed:

$E(Y | X, R = responded, Mode=telephone) = X\beta$;

$E(Y | X, R = non\_responded, Mode=telephone) = X\beta$;

$E(Y | X, R = responded, Mode=Mobile\_web) = X\beta+\gamma$;

$$E(Y | X, R = non\_responded, Mode=mobile\_web) = X\beta+\gamma,$$

where $Y$ is the patient experience score, $R$ is the response indicator and $X$ are controlling variables (gender, age group, field of care, hospital of origin), β are regression coefficients, and $\gamma$ represents the mode effect (i.e., the mean difference in scores between mobile web and telephone modes after adjusting for $X$).

In the delta-adjust method, we introduce a sensitivity parameter $\delta_{1}$ and $\delta_{2}$ to model potential

differences between respondents and non-respondents in each mode:

$E(Y | X, R = responded, Mode=telephone) = X\beta$;

$E(Y | X, R = non\_responded, Mode=telephone) = X\beta+\delta_{1}$;

$E(Y | X, R = responded, Mode=Mobile\_web) = X\beta+\gamma$;

$E(Y | X, R = non\_responded, Mode=mobile\_web) = X\beta+\gamma+\delta_{2}$.

In our sensitivity analysis, $\beta$ is fitted and the missing score ($Y$) is imputed while varying the pre-determined values of $\delta_{1}$ and $\delta_{2}$. Then, we explore how the (imputed) mean difference of two modes change according to the values of $\delta_{1}$ and $\delta_{2}$.

Supplementary Material B. Additional results

Tables S1-S7 reports Estimates mode effects on the total score and the scores for six subcategories.

*Notes for Tables S1-S7.*

Subgroup analysis results from a randomized controlled trial of 3,200 adult inpatients from four general hospitals in South Korea (October-November 2022), randomly allocated to telephone or mobile web survey modes. Response rates varied by subgroup, with overall completion of 878 participants (358 telephone, 520 mobile web). "Diff." represents the difference in mean scores (mobile web – telephone); negative values indicate lower scores in mobile web surveys. '*P_int*' indicates the *P* value for interaction between survey mode and subgroup variable, testing whether mode effects differ across subgroups. All scores are scaled to 100 points maximum. Unadjusted estimates are from complete-case analysis. IPW (inverse probability weighting) and MI (multiple imputation) adjusted estimates account for potential non-response under the Missing-at-Random (MAR) assumption. Subgroups analyzed include: gender (male/female), age groups (19-39, 40-59, 60-69, 70+), field of care (medical/surgical & others).

**Table S1.** Estimated mode effects on the total score.

|  |  | Telephone  Mean (SD) | Mobile Mean (SD) | Unadjusted diff.  (95% CI) |  | IPW–Adjusted diff.  (95% CI) |  | MI–Adjusted diff.  (95% CI) |  |
| --- | --- | --- | --- | --- | --- | --- | --- | --- | --- |
|  |  |  |  |  | *P* value |  | *P* value |  | *P* value |
| **Gender** | |  |  | (*P_int* = .92) |  | (*P_int* = .44) |  | (*P_int* = .72) |  |
|  | Male | 84.26 (14.46) | 80.87 (16.27) | –3.39 (–6.31, –0.47) | .02 | –3.28 (–6.21, –0.35) | .03 | –4.38 (–7.05, –1.72) | .004 |
|  | Female | 85.71 (14.20) | 82.10 (16.44) | –3.61 (–6.64, –0.58) | .02 | –4.92 (–7.83, –2.01) | <.001 | –4.79 (–8.03, –1.55) | .009 |
| **Age group** | |  |  | (*P_int* = .97) |  | (*P_int* = .58) |  | (*P_int* = .72) |  |
|  | 19–39 | 88.18 (12.69) | 84.10 (17.51) | –4.08 (–10.47, 2.31) | .21 | –2.81 (–8.82, 3.21) | .36 | –5.15 (–8.53, –1.77) | .003 |
|  | 40–59 | 86.39 (13.93) | 82.67 (15.63) | –3.72 (–7.07, –0.36) | .03 | –2.33 (–5.95, 1.29) | .21 | –4.38 (–7.88, –0.88) | .02 |
|  | 60–69 | 84.22 (14.36) | 79.35 (16.18) | –4.87 (–8.70, –1.03) | .01 | –5.49 (–9.40, –1.59) | .006 | –4.66 (–8.60, –0.72) | .03 |
|  | 70+ | 82.41 (15.22) | 78.68 (17.04) | –3.73 (–8.70, 1.25) | .14 | –5.34 (–9.24, –1.43) | .007 | –4.58 (–8.65, –0.52) | .03 |
| **Field of care** | |  |  | (*P_int* = >.99) |  | (*P_int* = .77) |  | (*P_int* = .85) |  |
|  | Medical | 84.79 (14.98) | 81.19 (16.57) | –3.60 (–7.15, –0.06) | .05 | –4.49 (–7.81, –1.18) | .008 | –4.44 (–7.43, –1.45) | .007 |
|  | Surgical & other | 84.98 (14.02) | 81.69 (16.25) | –3.30 (–5.90, –0.69) | .01 | –3.86 (–6.50, –1.22) | .004 | –4.69 (–7.80, –1.58) | .009 |

**Table S2.** Estimated mode effects on the score for Nurse domain.

|  |  | Telephone  Mean (SD) | Mobile Mean (SD) | Unadjusted diff.  (95% CI) |  | IPW–Adjusted diff.  (95% CI) |  | MI–Adjusted diff.  (95% CI) |  |
| --- | --- | --- | --- | --- | --- | --- | --- | --- | --- |
|  |  |  |  |  | *P* value |  | *P* value |  | *P* value |
| **Gender** | |  |  | (*P_int* = .81) |  | (*P_int* = .97) |  | (*P_int* = .71) |  |
|  | Male | 89.75 (14.51) | 86.15 (16.03) | –3.60 (–6.67, –0.54) | .02 | –4.40 (–7.46, –1.34) | .005 | –4.57 (–7.37, –1.76) | .004 |
|  | Female | 88.97 (16.03) | 85.90 (17.99) | –3.07 (–6.25, 0.11) | .06 | –4.49 (–7.53, –1.45) | .004 | –5.02 (–8.43, –1.61) | .009 |
| **Age group** | |  |  | (*P_int* = .49) |  | (*P_int* = .79) |  | (*P_int* = .47) |  |
|  | 19–39 | 96.00 (9.29) | 87.09 (18.46) | –8.91 (–15.69, –2.14) | .009 | –6.31 (–12.59, –0.02) | .05 | –6.47 (–10.03, –2.91) | <.001 |
|  | 40–59 | 91.98 (13.24) | 87.73 (15.86) | –4.25 (–7.75, –0.76) | .02 | –3.27 (–7.06, 0.51) | .09 | –4.70 (–8.39, –1.01) | .02 |
|  | 60–69 | 87.75 (15.64) | 84.11 (17.76) | –3.64 (–7.64, 0.37) | .07 | –3.92 (–8.00, 0.16) | .06 | –4.47 (–8.62, –0.32) | .04 |
|  | 70+ | 85.23 (17.65) | 82.72 (17.25) | –2.51 (–7.70, 2.68) | .34 | –5.54 (–9.63, –1.46) | .008 | –4.53 (–8.81, –0.25) | .04 |
| **Field of care** | |  |  | (*P_int* = .50) |  | (*P_int* = .78) |  | (*P_int* = .56) |  |
|  | Medical | 88.83 (16.26) | 86.47 (16.62) | –2.35 (–6.08, 1.37) | .22 | –4.05 (–7.52, –0.59) | .02 | –4.31 (–7.45, –1.17) | .01 |
|  | Surgical & other | 89.69 (14.65) | 85.76 (17.31) | –3.93 (–6.66, –1.20) | .005 | –4.69 (–7.44, –1.94) | <.001 | –5.11 (–8.39, –1.84) | .007 |

**Table S3.** Estimated mode effects on the score for the Doctor domain.

|  |  | Telephone  Mean (SD) | Mobile Mean (SD) | Unadjusted diff.  (95% CI) |  | IPW–Adjusted diff.  (95% CI) |  | MI–Adjusted diff.  (95% CI) |  |
| --- | --- | --- | --- | --- | --- | --- | --- | --- | --- |
|  |  |  |  |  | *P* value |  | *P* value |  | *P* value |
| **Gender** | |  |  | (*P_int* = .91) |  | (*P_int* = .92) |  | (*P_int* = .88) |  |
|  | Male | 81.92 (18.30) | 77.60 (20.11) | –4.32 (–7.97, –0.67) | .02 | –5.11 (–8.78, –1.43) | .006 | –5.10 (–8.42, –1.78) | .006 |
|  | Female | 83.22 (19.40) | 79.20 (19.77) | –4.02 (–7.81, –0.23) | .04 | –4.84 (–8.49, –1.19) | .009 | –5.32 (–9.35, –1.28) | .02 |
| **Age group** | |  |  | (*P_int* = .91) |  | (*P_int* = .43) |  | (*P_int* = .67) |  |
|  | 19–39 | 83.65 (20.89) | 81.13 (22.12) | –2.52 (–10.55, 5.50) | .54 | –2.24 (–9.78, 5.31) | .56 | –5.42 (–9.64, –1.21) | .01 |
|  | 40–59 | 83.87 (18.14) | 79.55 (19.70) | –4.32 (–8.53, –0.11) | .04 | –2.67 (–7.21, 1.88) | .25 | –5.00 (–9.36, –0.63) | .03 |
|  | 60–69 | 81.71 (19.01) | 76.52 (18.41) | –5.19 (–10.00, –0.37) | .04 | –6.43 (–11.33, –1.53) | .01 | –5.19 (–10.10, –0.28) | .04 |
|  | 70+ | 81.11 (18.84) | 75.12 (20.46) | –5.99 (–12.24, 0.25) | .06 | –7.35 (–12.25, –2.46) | .003 | –5.48 (–10.54, –0.42) | .04 |
| **Field of care** | |  |  | (*P_int* = .60) |  | (*P_int* = .43) |  | (*P_int* = .97) |  |
|  | Medical | 83.14 (19.50) | 78.09 (20.61) | –5.05 (–9.49, –0.61) | .03 | –6.27 (–10.43, –2.12) | .003 | –5.24 (–8.96, –1.52) | .01 |
|  | Surgical & other | 82.18 (18.44) | 78.62 (19.56) | –3.56 (–6.82, –0.30) | .03 | –4.15 (–7.46, –0.84) | .01 | –5.19 (–9.06, –1.32) | .02 |

**Table S4.** Estimated mode effects on the score for the Medication and treatment domain.

|  |  | Telephone  Mean (SD) | Mobile Mean (SD) | Unadjusted diff.  (95% CI) |  | IPW–Adjusted diff.  (95% CI) |  | MI–Adjusted diff.  (95% CI) |  |
| --- | --- | --- | --- | --- | --- | --- | --- | --- | --- |
|  |  |  |  |  | *P* value |  | *P* value |  | *P* value |
| **Gender** | |  |  | (*P_int* = .77) |  | (*P_int* = .36) |  | (*P_int* = .63) |  |
|  | Male | 84.17 (16.98) | 81.32 (18.73) | –2.85 (–6.22, 0.52) | .10 | –2.79 (–6.21, 0.62) | .11 | –4.16 (–7.23, –1.08) | .01 |
|  | Female | 86.17 (16.33) | 82.59 (18.83) | –3.58 (–7.08, –0.08) | .04 | –5.04 (–8.43, –1.65) | .004 | –4.79 (–8.52, –1.06) | .02 |
| **Age group** | |  |  | (*P_int* = .84) |  | (*P_int* = .47) |  | (*P_int* = .60) |  |
|  | 19–39 | 89.73 (14.54) | 84.59 (20.00) | –5.14 (–12.52, 2.24) | .17 | –3.08 (–10.09, 3.94) | .39 | –5.41 (–9.31, –1.51) | .007 |
|  | 40–59 | 86.22 (16.40) | 83.42 (17.89) | –2.80 (–6.67, 1.07) | .16 | –1.40 (–5.62, 2.83) | .52 | –4.06 (–8.10, –0.02) | .05 |
|  | 60–69 | 84.73 (15.89) | 79.46 (18.05) | –5.27 (–9.70, –0.84) | .02 | –6.03 (–10.59, –1.47) | .010 | –4.68 (–9.22, –0.13) | .04 |
|  | 70+ | 82.07 (18.59) | 78.95 (20.92) | –3.12 (–8.87, 2.62) | .29 | –5.12 (–9.68, –0.57) | .03 | –4.43 (–9.11, 0.26) | .06 |
| **Field of care** | |  |  | (*P_int* = .96) |  | (*P_int* = .98) |  | (*P_int* = .80) |  |
|  | Medical | 84.60 (17.75) | 81.59 (19.43) | –3.01 (–7.10, 1.09) | .15 | –3.96 (–7.83, –0.10) | .04 | –4.25 (–7.69, –0.81) | .02 |
|  | Surgical & other | 85.33 (16.15) | 82.20 (18.42) | –3.13 (–6.14, –0.13) | .04 | –3.90 (–6.98, –0.83) | .01 | –4.63 (–8.21, –1.04) | .02 |

**Table S5.** Estimated mode effects on the score for the Hospital environment domain.

|  |  | Telephone  Mean (SD) | Mobile Mean (SD) | Unadjusted diff.  (95% CI) |  | IPW–Adjusted diff.  (95% CI) |  | MI–Adjusted diff.  (95% CI) |  |
| --- | --- | --- | --- | --- | --- | --- | --- | --- | --- |
|  |  |  |  |  | *P* value |  | *P* value |  | *P* value |
| **Gender** | |  |  | (*P_int* = .56) |  | (*P_int* = .10) |  | (*P_int* = .59) |  |
|  | Male | 84.62 (19.19) | 78.60 (21.54) | –6.02 (–9.74, –2.30) | .002 | –6.17 (–9.84, –2.50) | .001 | –7.42 (–10.79, –4.05) | <.001 |
|  | Female | 87.92 (17.00) | 80.32 (20.19) | –7.60 (–11.46, –3.74) | <.001 | –10.51 (–14.16, –6.87) | <.001 | –8.19 (–12.28, –4.10) | .002 |
| **Age group** | |  |  | (*P_int* = .64) |  | (*P_int* = .10) |  | (*P_int* = .65) |  |
|  | 19–39 | 88.26 (16.68) | 85.19 (22.08) | –3.07 (–11.22, 5.08) | .46 | –4.48 (–12.02, 3.05) | .24 | –7.58 (–11.85, –3.31) | <.001 |
|  | 40–59 | 86.95 (17.87) | 80.17 (19.96) | –6.78 (–11.05, –2.50) | .002 | –5.18 (–9.72, –0.64) | .03 | –7.49 (–11.91, –3.06) | .004 |
|  | 60–69 | 85.02 (18.05) | 76.06 (20.26) | –8.96 (–13.85, –4.06) | <.001 | –9.16 (–14.06, –4.27) | <.001 | –8.13 (–13.11, –3.15) | .005 |
|  | 70+ | 85.61 (19.91) | 77.10 (22.10) | –8.52 (–14.86, –2.18) | .009 | –12.88 (–17.77, –7.99) | <.001 | –8.08 (–13.22, –2.95) | .006 |
| **Field of care** | |  |  | (*P_int* = .88) |  | (*P_int* = .71) |  | (*P_int* = .95) |  |
|  | Medical | 85.18 (19.14) | 78.31 (21.53) | –6.88 (–11.40, –2.35) | .003 | –8.97 (–13.13, –4.81) | <.001 | –7.75 (–11.52, –3.98) | <.001 |
|  | Surgical & other | 86.62 (17.83) | 80.16 (20.45) | –6.46 (–9.77, –3.14) | <.001 | –7.97 (–11.28, –4.66) | <.001 | –7.85 (–11.78, –3.92) | .002 |

**Table S6.** Estimated mode effects on the score for the Patient’s rights domain.

|  |  | Telephone  Mean (SD) | Mobile Mean (SD) | Unadjusted diff.  (95% CI) |  | IPW–Adjusted diff.  (95% CI) |  | MI–Adjusted diff.  (95% CI) |  |
| --- | --- | --- | --- | --- | --- | --- | --- | --- | --- |
|  |  |  |  |  | *P* value |  | *P* value |  | *P* value |
| **Gender** | |  |  | (*P_int* = .64) |  | (*P_int* = .64) |  | (*P_int* = .66) |  |
|  | Male | 80.15 (19.55) | 77.38 (18.76) | –2.77 (–6.35, 0.80) | .13 | –1.99 (–5.62, 1.65) | .28 | –2.46 (–7.46, 2.54) | .28 |
|  | Female | 81.59 (20.18) | 80.05 (18.14) | –1.55 (–5.25, 2.16) | .41 | –3.20 (–6.81, 0.40) | .08 | –3.37 (–7.43, 0.68) | .09 |
| **Age group** | |  |  | (*P_int* = .62) |  | (*P_int* = .58) |  | (*P_int* = .62) |  |
|  | 19–39 | 82.31 (18.22) | 84.06 (17.95) | 1.75 (–6.15, 9.64) | .66 | 2.27 (–5.21, 9.75) | .55 | –1.80 (–10.43, 6.82) | .64 |
|  | 40–59 | 83.65 (18.46) | 79.37 (18.34) | –4.28 (–8.37, –0.19) | .04 | –2.75 (–7.24, 1.75) | .23 | –3.48 (–9.24, 2.27) | .19 |
|  | 60–69 | 79.41 (21.24) | 76.31 (18.36) | –3.10 (–7.78, 1.59) | .19 | –3.95 (–8.80, 0.90) | .11 | –3.05 (–7.50, 1.40) | .16 |
|  | 70+ | 77.85 (20.10) | 75.09 (18.50) | –2.75 (–8.82, 3.32) | .37 | –3.13 (–7.98, 1.72) | .21 | –2.88 (–5.96, 0.21) | .07 |
| **Field of care** | |  |  | (*P_int* = .70) |  | (*P_int* = .90) |  | (*P_int* = .86) |  |
|  | Medical | 81.12 (20.74) | 78.39 (18.57) | –2.74 (–7.08, 1.60) | .22 | –2.81 (–6.92, 1.30) | .18 | –3.14 (–8.44, 2.15) | .20 |
|  | Surgical & other | 80.64 (19.37) | 78.97 (18.44) | –1.67 (–4.86, 1.52) | .30 | –2.47 (–5.74, 0.81) | .14 | –2.78 (–6.57, 1.01) | .13 |

**Table S7.** Estimated mode effects on the score for the Overall rating domain.

|  |  | Telephone  Mean (SD) | Mobile Mean (SD) | Unadjusted diff.  (95% CI) |  | IPW–Adjusted diff.  (95% CI) |  | MI–Adjusted diff.  (95% CI) |  |
| --- | --- | --- | --- | --- | --- | --- | --- | --- | --- |
|  |  |  |  |  | *P* value |  | *P* value |  | *P* value |
| **Gender** | |  |  | (*P_int* = .42) |  | (*P_int* = .11) |  | (*P_int* = .93) |  |
|  | Male | 84.15 (17.32) | 83.96 (18.67) | –0.19 (–3.62, 3.23) | .91 | 2.45 (–1.01, 5.91) | .17 | –0.69 (–3.37, 1.99) | .59 |
|  | Female | 86.51 (16.44) | 84.28 (19.52) | –2.23 (–5.80, 1.33) | .22 | –1.52 (–4.95, 1.92) | .39 | –0.85 (–5.21, 3.50) | .66 |
| **Age group** | |  |  | (*P_int* = .31) |  | (*P_int* = .11) |  | (*P_int* = .23) |  |
|  | 19–39 | 85.00 (18.21) | 81.67 (22.98) | –3.33 (–10.87, 4.20) | .39 | –0.97 (–8.07, 6.14) | .79 | –1.75 (–10.75, 7.25) | .66 |
|  | 40–59 | 83.87 (18.19) | 85.24 (17.71) | 1.37 (–2.58, 5.32) | .50 | 3.14 (–1.14, 7.42) | .15 | –0.34 (–4.50, 3.81) | .86 |
|  | 60–69 | 87.46 (15.22) | 83.50 (19.25) | –3.95 (–8.48, 0.57) | .09 | –4.01 (–8.62, 0.61) | .09 | –0.93 (–7.20, 5.34) | .73 |
|  | 70+ | 84.27 (16.74) | 84.63 (18.19) | 0.36 (–5.51, 6.24) | .90 | 2.38 (–2.23, 7.00) | .31 | –0.74 (–4.91, 3.43) | .70 |
| **Field of care** | |  |  | (*P_int* = .61) |  | (*P_int* = .49) |  | (*P_int* = .88) |  |
|  | Medical | 85.41 (16.41) | 83.45 (20.17) | –1.96 (–6.13, 2.21) | .36 | –0.62 (–4.54, 3.30) | .76 | –0.94 (–3.96, 2.08) | .52 |
|  | Surgical & other | 85.13 (17.25) | 84.50 (18.49) | –0.62 (–3.68, 2.43) | .69 | 1.13 (–1.99, 4.25) | .48 | –0.67 (–4.64, 3.31) | .70 |
