# Supplementary material for: Mode Effects Between Mobile Web and Telephone Surveys on Patient Experience Scores in South Korea: Secondary Analysis of a Randomized Controlled Trial Under Various Missingness Scenarios
Source: J Med Internet Res. 2026 May 14;28:e79398. doi: 10.2196/79398 (PMC13175444; doi:10.2196/79398)
Supplement: Checklist 3 [file jmir-v28-e79398-s004.pdf]

Items to include when reporting a randomized trial in a journal or conference abstract

Item	Description	Reported on line number
Title	Identification of the study as randomized	1-4
Authors *	Contact details for the corresponding author	6-7, 15-23
Trial design	Description of the trial design (e.g. parallel, cluster, non-inferiority)	40-41
Methods		
Participants	Eligibility criteria for participants and the settings where the data were collected	41-43
Interventions	Interventions intended for each group	43-44
Objective	Specific objective or hypothesis	37-39
Outcome	Clearly defined primary outcome for this report	38-39, 56-59
Randomization	How participants were allocated to interventions	43-45
Blinding (masking)	Whether or not participants, care givers, and those assessing the outcomes were blinded to group assignment	45-46
Results		
Numbers randomized	Number of participants randomized to each group	53
Recruitment	Trial status	40-43
Numbers analysed	Number of participants analysed in each group	53-54
Outcome	For the primary outcome, a result for each group and the estimated effect size and its precision	38-39, 56-59
Harms	Important adverse events or side effects	55-56
Conclusions	General interpretation of the results	63-70
Trial registration	Registration number and name of trial register	72-75
Funding	Source of funding	77-78

**this item is specific to conference abstracts*
